# SnO_2_/Pt Thin Film Laser Ablated Gas Sensor Array

**DOI:** 10.3390/s110807724

**Published:** 2011-08-05

**Authors:** Mohammad Hadi Shahrokh Abadi, Mohd Nizar Hamidon, Abdul Halim Shaari, Norhafizah Abdullah, Rahman Wagiran

**Affiliations:** 1 Electrical and Electronic Department, Engineering Faculty, Universiti Putra Malaysia 43400, Serdang, Selangor, Malaysia; E-Mails: mnh@eng.upm.edu.my (M.N.H.); rwagiran@eng.upm.edu.my (R.W.); 2 Physics Department, Science Faculty, Universiti Putra Malaysia 43400, Serdang, Selangor, Malaysia; E-Mail: ahalim@fsas.upm.edu.my (A.H.S.); 3 Department of Chemical & Environmental Engineering, Engineering Faculty, Universiti Putra Malaysia 43400, Serdang, Selangor, Malaysia; E-Mail: fizah@eng.upm.edu.my (N.A.)

**Keywords:** gas sensor, thin film, pulse laser ablation deposition, screen printing, tin oxide, platinum

## Abstract

A gas sensor array was developed in a 10 × 10 mm^2^ space using Screen Printing and Pulse Laser Ablation Deposition (PLAD) techniques. Heater, electrode, and an insulator interlayer were printed using the screen printing method on an alumina substrate, while tin oxide and platinum films, as sensing and catalyst layers, were deposited on the electrode at room temperature using the PLAD method, respectively. To ablate SnO_2_ and Pt targets, depositions were achieved by using a 1,064 nm Nd-YAG laser, with a power of 0.7 J/s, at different deposition times of 2, 5 and 10 min, in an atmosphere containing 0.04 mbar (4 kPa) of O_2_. A range of spectroscopic diffraction and real space imaging techniques, SEM, EDX, XRD, and AFM were used in order to characterize the surface morphology, structure, and composition of the films. Measurement on the array shows sensitivity to some solvent and wood smoke can be achieved with short response and recovery times.

## 1. Introduction

An array gas sensor is comprised of two or more sensors on a nonconductive substrate, having individual sensor elements, formed by semiconductor oxides. Since a single gas sensor is only sensitive to one chemical contaminant and cannot produce all the information for many chemical species, an array gas sensor is necessary to detect the several contaminants in a single device, leading to development of so-called electronic noses.

A variety of different techniques and materials have been employed to fabricate array gas sensors. Soft lithography micromolding in capillaries has been used by Heule *et al*. [1] to fabricate twelve miniaturized gas sensors of nanoparticulate tin oxide as an array on a single microhotplate to detect carbon monoxide. Ha *et al*., developed a sensor array device consists of 16 separate sensors; each sensor equipped with an interdigitated electrode and integrated Pt microheater using micromachined wet etching well [2]. A monolithic QCM sensor array for gas detections based-on MEMS was developed by Xiaoxia *et al*. [3]. A reactive r.f. sputtering magnetron system has been used by Penza *et al*. [4] to deposit a sensitive WO_3_ thin layer onto alumina substrate as an array gas sensor including four individual sensors. The sensors have been differently surface modified by four metal catalysts as Pd, Au, Bi, Sb in order to integrate bi-layers with different chemical sensitivities, operating at 180 °C, and principal component analysis (PCA) has been used in order to classify and identify different classes of target gases. Although the sensors array can provide a specific and unique response pattern for different individual chemical species or mixtures of species, however, a pattern recognition analysis needs to be applied to make an effective approach for enhancement the selectivity and range of practical applications of chemical sensors systems [5]. Yaowu *et al*., have reported a micromachining technique to fabricate eight individual gas sensors in form of an array onto an n-type silicon wafer [6]. They deposited a composition of SnO_2_ and Ti_2_O_3_ as sensitive layer by electron beam evaporation using a metal mask. Their results showed a relatively high sensitivity and moderate selectivity to alcohol type. Adami *et al*., have used low pressure chemical vapour deposition (LPCVD) to deposit all layer of an array gas sensor, while r.f. sputtering has been used for deposition tungsten oxide, as sensitive layer [7,8].

The fabrication process of an array gas sensor based-on above mentioned techniques, particularly for the micromachining of Si, has very promising because these fabrication procedure can be compatible with the integrated circuit (IC) process, and make it possible to integrate sensors with signal conditioning and drive circuit together, but at the same time, different processes would be needed to deposit individual layers: e.g., the process of chemical wetting involves a multi-stage process demanding long duration [9,10]. Chemical vapor deposition (CVD) suffers from complex processes, temperatures of 600 °C and above needed to grow epitaxial films, and requires starter materials with high vapor pressure which are often hazardous and extremely toxic [11,12].

Another method for deposition a sensitive thin layer is pulse laser ablation deposition (PLAD), with many promises for deposition different thin layers in sandwich form based-on different targets, which can operated under any ambient gas (pressure range 0–1 Torr), with ease of thickness control, low substrate temperature, and reasonable deposition rate [13–15].

In the current work, a gas sensor array, including four individual sensors is developed. Screen printing technique is employed to fabricate the heater and electrode parts, while the pulse laser ablation deposition (PLAD) is used to deposit the sensitive (SnO_2_) and catalyst (Pt) layers.

## 2. Experimental

**Figure 1.**
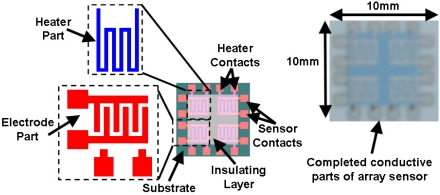
Electric parts of array gas sensor.

The array gas sensor was fabricated using screen printing and laser ablation techniques. Heater, electrode, and an inter-insulator layer of each sensor were fabricated in an area of 4.2 × 4.2 mm^2^ using the screen printing method. Platinum paste (ESL-5545, Electrosience Lab Inc., UK) was used to print four individual heaters on the alumina substrate (ADS996-STD 60 × 50 × 0.25, CoorsTek, USA) in an area of 10 × 10 mm^2^ followed by thermal curing. An insulating paste was prepared using mixture of alumina powder, glass powder and an organic vehicle, printed on the heater surface, followed by drying and firing process. Electrode and contacts patterns were carefully aligned and printed on the insulating layer using silver paste (ESL-9912A, Electrosience Lab Inc., UK) and thermal curing was applied. Figure 1 shows the layers of array before deposition of sensing layer.

**Figure 2.**
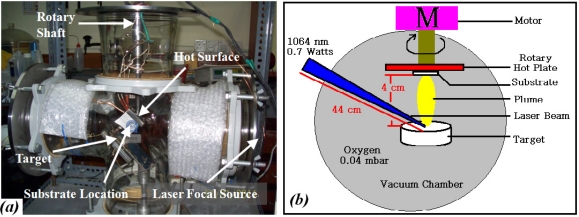
**(a)** Actual system and **(b)** Schematic diagram of PLA chamber and a solid target with laser beam and motor.

Tin dioxide (SnO_2_) and platinum (Pt) in form of pellets were used as the targets in the PLAD chamber, to deposit the sensitive and catalyst layers, respectively. The powders were calcinated at 950 °C for 4 h, and formed in a pellet of 10 × 1 mm^2^ using a hydraulic press, set at 11 (SnO_2_) and 7.5 (Pt) tons for 15 min, then the pellets were sintered at 800 °C for 1 h (5 °C min^−1^). Structural and elemental properties of the pellets were determined by XRD. Quanta System, HYL 101E Nd-YAG laser (λ = 1,064 nm, P = 0.7 watts) laser ablation machine was used to deposit the SnO_2_ on the electrode area, and then the Pt was deposited on the SnO_2_ layer. The distance of pellets to substrate and the distance of laser source to the pellet were kept at 4 cm and at 44 cm, respectively. Oxygen at 4 × 10^−2^ mbar was used as ambient gas and the deposition was carried out at room temperature. Figure 2 schematically illustrates the chamber.

**Figure 3.**
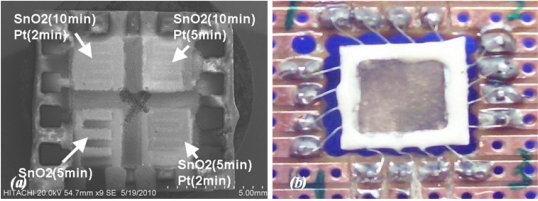
**(a)** SEM micrograph of array with different deposition time of SnO_2_ and Pt; and **(b)** wire-bonded array in a 16-pins-quad carrier.

The SnO_2_ sensitive layer was formed at different deposition times of 5 and 10 min on each pair of sensors, respectively. Three sensors were covered with a plume of Pt at different deposition times of 2 and 5 min to form the catalyst layer, whilst the last one was abandoned. Afterward, films were fired at peak temperature of 450 °C (2 °C min^−1^) for 1 h to develop the structural and electrical properties of the films, then device was slowly cold down to room temperature. Figure 3(a) shows SEM micrograph of array sensor. A carrier was developed using PCB and the array was soldered to the carrier. Soldered wires on the array were covered using silicate-base cement (Sauereisen 33S-Ellsworth Adhesives-USA) to prevent wire separation and lead migration at working temperature above 150 °C.

## 3. Characterization of the Gas Sensor Array

### 3.1. X-Ray Diffraction (XRD) Analysis

**Figure 4.**
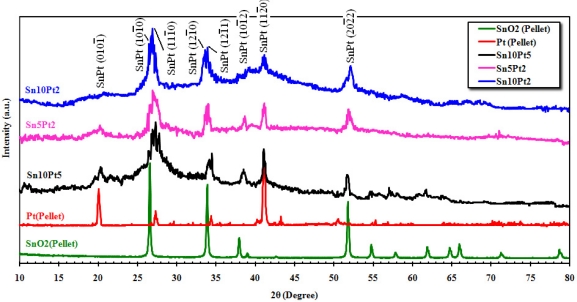
X-ray diffraction analysis of film’s surface.

Figure 4 shows the XRD spectrum of the SnO_2_ and Pt pellets, and film’s surfaces matched with JCPDS files. From the pattern of SnO_2_ pellet, broad diffraction peaks of the tetragonal SnO_2_ can be seen at about 2θ = 26.43°, 33.72°, 37.80°, 39.04°, 51.61°, 54.61°, 57.69°, 61.73°, 64.61°, 65.81°, 71.13°, and 78.58° attributed to (110), (101), (200), (210), (211), (220), (002), (202), (310), (112), (301), and (321) planes, respectively. The maximum intensity of Pt pellet was observed at 2θ = 40.58° attributed to (120) plane, while the other significant peaks are located at about 20.12°, 27.34°, 34.44°, 42.72°, and 50.61° attributed to (001), (110), (111), (120), and (220) planes. The patterns indicate the presence of metallic platinum that is present in the face-centered cubic form. The large and very broad peak at about 2θ = 26.43° is due to the anomalous scattering from the metallic effect of Pt on SnO_2_. This scattering was observed for all alloy phases. The XRD pattern also shows that the wideness is alerted due to amount of crystalline platinum. The average crystallite size of the above mentioned peaks was determined from the Scherrer Equation (Scherrer Constant *K* = 0.94 and *λ* = 1.542 Å), described elsewhere [16,17], is about 15 nm. Since for a welfare particle, there would be 2 to 10 scattered crystallites, therefore an average size of about 80 nm can be determined for the particles.

### 3.2. Surface Analysis of Deposited Film

**Figure 5.**
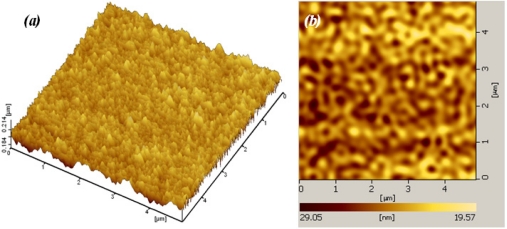
AFM micrograph of **(a)** the surface of SnO_2_ pellet; **(b)** the surface of Sn10Pt2 film.

The surface morphology of the film was studied using both Atomic Force Microscopy (AFM) and Scanning Electron Microscope (SEM). Figure 5 shows an AFM micrograph of the SnO_2_ pellet and surface of Sn10Pt2 film, taken over an area of 5 × 5 μm^2^. Analysis was run under two channel simulated mode, channel A for topology (Z sensing) and channel B for deflection. From grain size analysis of AFM and for the given area of the pellet surface (Figure 5(a)), the average grain size of 2.432E + 4 nm^2^ ± 5% and average diameter of 176 ± 35 nm for the grains was observed. AFM analysis of the deposited film on the surface of Sn10Pt2 sample (Figure 5(b)) shows an average of particle size less than 20 nm. Normally, deposition of SnO_2_ using PLAD is carried out on a hot substrate (≈ 300 °C) by control of the background oxygen pressure to produce fine particle sizes less than 10 nm. Above this temperature causes the crystal size to be more than doubled. However, fully crystalline SnO_2_ film is obtained at temperatures ≥150 °C. In our study, the films were deposited at room temperature, whereas this condition produces mainly amorphous phases, resulted in higher porosity.

**Figure 6.**
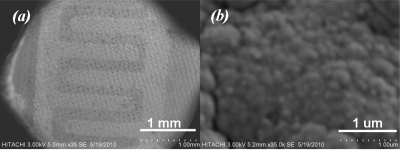
**(a)** Effect of screen lift up; and **(b)** formation of agglomerated SnO_2_ clusters.

**Figure 7.**
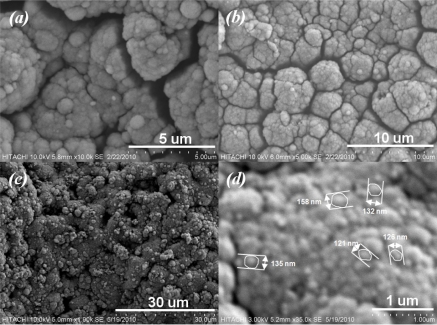
SEM micrograph of sensor surface.

Figure 6 shows SEM micrographs taken from surface of sensitive area. The unevenness of the film surface in Figure 6(a) is due to the printing screen of the insulating and electrode layers. Formation of SnO_2_ agglomerates can be seen from Figure 6(b). In addition to the AFM result, a porous surface of the sensitive film can be seen from Figure 7. The formation of continuous, finely agglomerated, closed and compact particles of film (Figure 7(a,b)) which are crack free (Figure 7(c)) is evident from this SEM images. Grain size of the films estimated from the SEM image (Figure 7(d)) lies well below 150 nm and thus supports the findings from X-ray diffraction pattern and those from AFM results.

**Figure 8.**
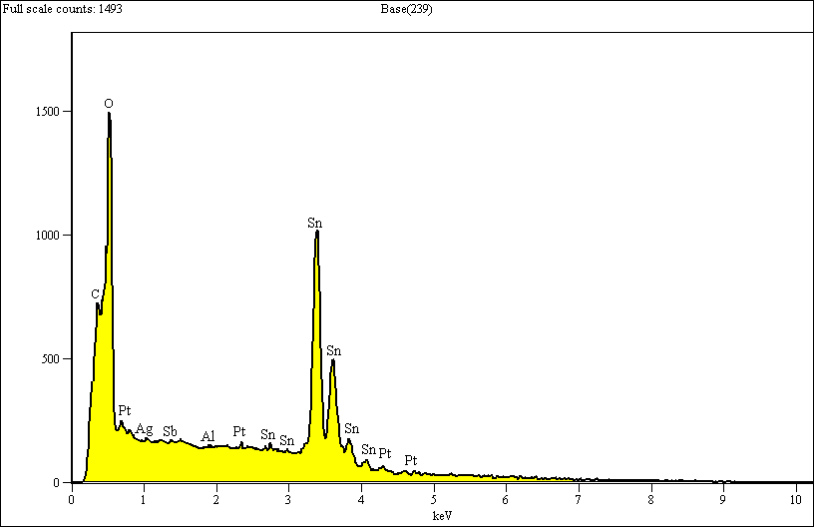
Elemental result of Sn10Pt5 film surface.

Figure 8 shows the EDX analysis of a selected area of 10 × 10 μm^−2^ from the surface of the sensor with 10 min SnO_2_ deposition followed by 5 min Pt. The elemental analysis shows an elemental composition of Al, C, O, Pt, Sn, and Sb. The presence of carbon is due to the use of carbon tape to attach the samples to the SEM platform. The presence of Al, Ag, and Sb might be due to some penetration of components of the insulating and electrode layers into the film’s surface during the firing process.

### 3.3. Gas Sensing Performance of Array

**Figure 9.**
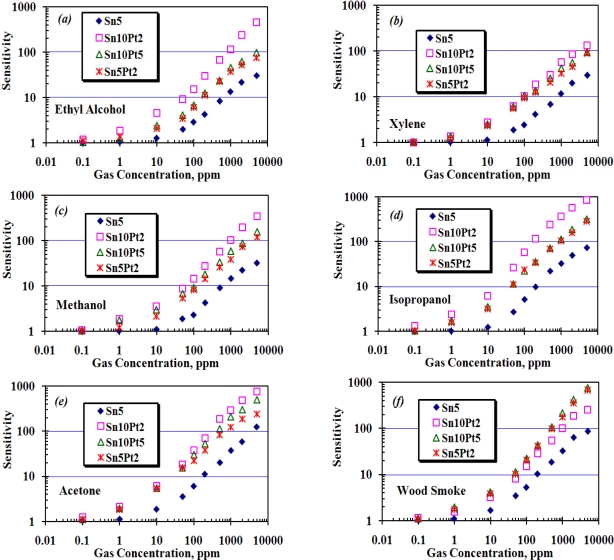
Sensitivity of array in presence of different concentration of: **(a)** ethyl alcohol; **(b)** xylene; **(c)** methanol; **(d)** isopropanol; **(e)** acetone; and **(f)** wood smoke. The number after the element’s name indicates deposition time of the element in minutes.

The array was exposed to different concentrations of wood smoke, ethyl alcohol, *m*-xylene, methanol, isopropanol, and acetone up to 5,000 ppm. During the experiment, working temperature of each sensor, ambient humidity and temperature were kept at 200 ± 10 °C, 55 ± 5% and 25 ± 3 °C, respectively. A base line for each sensor was determined by measuring its resistance in presence of fresh air (*R_a_*), and then the resistance of each sensor, *R_g_*, was measured in presence of the applied gas. To study the sensitivity of each sensor in the array, *R_g_* was normalized by *R_a_* as shown in Figure 9. In this figure, the X-axis shows the concentration of applied gas from 0.1 ppm to 5,000 ppm, which is represented on a logarithmic scale and the Y-axis shows the sensitivity, which is calculated as the ratio of *R_a_*/*R_g_*.

**Figure 10.**
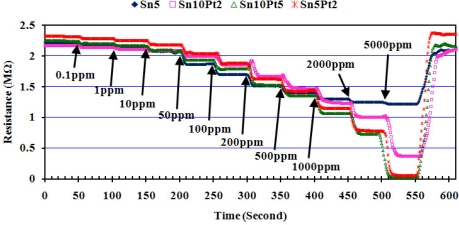
Resistance of array, working at *T* = 200 ± 10 °C and *RH* = 55 ± 5%, to different concentration of wood smoke.

The enhanced sensitivity of nanocrystalline SnO_2_ thin film is attributed to two aspects [18] plus the thickness of the SnO_2_ layer itself, studied by Tricoli *et al.* [19]. In the first aspect, decreasing the particle size leads to better sensitivity by increasing the surface to volume ratio (inversely proportional to the particle size). In the second aspect, better porosity of the gas sensing film has an impact on gas sensing behavior. Increasing film thickness decreases the sensor response as the fraction of material involved in the interaction with the analyte is monotonously decreased. The AFM analysis in Figure 5(b) shows a thickness of almost 50 nm of the dense film, with an average particle size of <20 nm for the given area. As the film thickness (50 nm) and particle size (20 nm) were both considerably above twice the Debye length of SnO_2_ particles (≈6 nm at room temperature [20]), the sensor has only moderate sensitivity at room temperature. If the particle concentration of SnO_2_ remains constant, the Debye length of SnO_2_ particles then would be only depend on the square root of temperature, *T*^1/2^, and so the maximum sensitivity of the sensor will be achieved at elevated temperatures. At this point, we set the operating temperature of the array at about 200 °C, and the array was consecutively exposed to the above mentioned species in form of either liquid or gas at quantities required to create concentrations of 0.1, 1, 10, 50, 100, 200, 500, 1,000, 2,000, and 5,000 ppm. The resistance of each sensor then was measured and recorded for further processing and sensitivity calculation. Figure 10 shows how the array responds to consecutively injection of wood smoke. Pt loaded sensors show to be highly sensitive compared to Sn5, moreover, the sensitivity is effectively affected by the ratio of deposition time of Sn:Pt.

Figure 9 shows the sensitivity of the array to the applied species. In the case of ethyl alcohol, xylene, methanol, isopropanol, and wood smoke, Sn10Pt5 and Sn5Pt2 show almost the same sensitivity to the applied species, while the Sn10Pt2 shows higher sensitivity than the others, except in the case of wood smoke. In the case of Acetone (Figure 9(e)), significant difference between the sensitivity of the sensors, particularly at 100 ppm of applied species and above, can be observed. The maximum sensitivity of the array was observed in presence of 5,000 ppm isopropanol and is attributed to Sn10Pt2. The maximum sensing values to 10 ppm ethyl alcohol, xylene, methanol, isopropanol, acetone, and wood smoke, detected by Sn10Pt2, were 4.5, 2.7, 3.5, 6.1, 6.1, and 3.2, respectively, whilst the sensing values (*R_a_/R_g_*) detected by Sn10Pt5 were 2.4, 2.5, 3.1, 3.5, 5.4, and 4.4. At higher concentration of the applied species, greater differences of the sensing values can be observed. In 1,000 ppm of applied species, the Sn10Pt2 shows a remarkable sensing value in presence of all species, except for the wood smoke, compared to the sensing value of the other sensors of the array. In the presence of solvents, what is detected by the array is a decrease of the band intensity ascribed to surface of hydroxyl groups including hydrogen, water, and oxygen species and an increase of bands assigned to hydrated proton species, whilst the band intensity of the SnO_2_ layer is attributed to CO and CO_2_ species contained in the wood smoke. Moreover, the SnO_2_ layer is modified by Pt nanocrystal grain boundaries and gives complex responses due to electron trap states formed at the interfaces of Pt and Sn grains. Higher concentration of Pt nanocrystals into the Sn sites makes a better trap for the CO and CO_2_ species, resulted in greater sensing values especially at higher concentration of applied CO and CO_2_.

**Figure 11.**
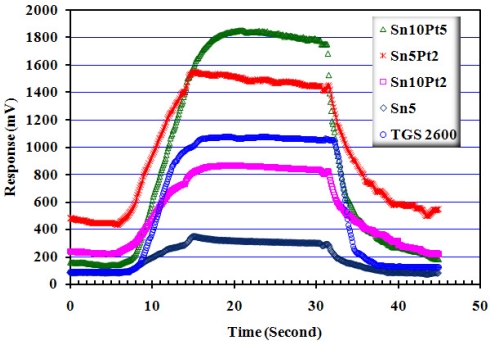
Response of array, working at *T* = 200 ± 10 °C and *RH* = 55 ± 5%, to 1,000 ppm wood smoke. For comparison, response of a Taguchi air sensor, TGS 2600, has been given.

Figure 11 shows how the array responds to 1,000 ppm wood smoke. An air quality sensor, TGS 2600, was used for comparison. The response time of each sensor is determined as the interval time between 10 to 90 percent of sensor signal. The minimum response time was observed is belong to Sn10Pt2 which is ≤6.5 s while the Sn10Pt5 shows the maximum signal. Detail of the response times to the 1,000 ppm of the applied species is given in Table 1.

## 4. Conclusions

An array gas sensor based-on thick film and laser ablation techniques was developed and the sensitivity of the array to wood smoke, ethyl alcohol, *m*-xylene, methanol, isopropanol, and acetone was measured. The array is moderately sensitive to sub ppm of the applied species and shows a great sensitivity to the applied gases at concentrations above 10 ppm with a response time below than 10 s. Measurements and modifications are necessary to eliminate or minimize the cross sensitivities to the other contaminants. Deposition of different noble metal, e.g., Au, Ag, and Pd, can promote the sensitivity of the array to different target gases.

## Figures and Tables

**Table 1.** Response time of array to 1,000 ppm methanol, isopropanol, acetone, and wood smoke.

***Sensor Code***	***Deposition Time, min***			***Response Time, sec***		

	***SnO****_2_*	***Pt***	**methanol**	**isopropanol**	**acetone**	**wood smoke**
Sn5	5	---	14.7	11.1	10.4	7.8
Sn5Pt2	5	2	12.5	7.4	7.6	6.3
Sn10Pt2	10	2	12.2	8.3	7.8	6.1
Sn10Pt5	10	5	10.1	5.2	4.8	7.2
